# Resolvin E1 reduces hepatic fibrosis in mice with *Schistosoma japonicum* infection

**DOI:** 10.3892/etm.2014.1641

**Published:** 2014-03-27

**Authors:** WENHONG QIU, KAIWEN GUO, LUYANG YI, YELI GONG, LIXIA HUANG, WEI ZHONG

**Affiliations:** 1 Department of Immunology, School of Medicine, Jianghan University, Wuhan, Hubei 430056, P.R. China; 2 Department of Immunology, Wuhan University of Science and Technology, Wuhan, Hubei 430080, P.R. China

**Keywords:** resolvin E1, hepatic fibrosis, anti-inflammatory, immune adjustment, infection, *Schistosoma japonicum*

## Abstract

The aim of this study was to investigate whether resolvin E1 (RvE1) protects against hepatic fibrosis in a murine model of liver fibrosis induced by *Schistosoma japonicum* infection. A total of 30 pathogen-free Kunming mice were randomly and equally divided into three groups: Control (uninfected, untreated), model (infected, untreated) and RvE1 intervention (infected, RvE1-treated; 100 ng daily). The mice were infected with *Schistosoma japonicum* by inoculating the abdominal skin with 20±2 cercariae to induce models of liver fibrosis. The area and numbers of the granulomas in the livers were assessed through histopathology after 70 days of treatment. The levels of tumor necrosis factor (TNF)-α and interferon (IFN)-γ were evaluated in the serum by enzyme-linked immunosorbent assay (ELISA). The expression levels of TNF-α were detected in the hepatic tissue by reverse transcription-polymerase chain reaction and western blot analysis. The activity levels of alanine aminotransferase and aspartate aminotransferase were determined in the serum by ELISA. The expression levels of laminin (LN), hyaluronic acid (HA), procollagen type III (PC-III) and type IV collagen (IV-C) were detected in the serum by radioimmunoassays. The results revealed that the mean area of the granulomas was smaller in the RvE1 intervention group compared with that in the model group. Following RvE1 treatment, the serum levels of TNF-α were lower than those in the model group, while the serum levels of IFN-γ were higher compared with those in the model group. The expression levels of TNF-α were lower in the hepatic tissue following RvE1 treatment compared with those in the model group. The indicators of liver fibrosis, the levels of LN, HA, PC-III and IV-C in the serum, were lower following RvE1 treatment than those in the model group. In conclusion, RvE1 treatment may reduce the growth of granulomas, thereby slowing the process of hepatic fibrosis, and this effect may be the result of anti-inflammatory and immune system adjustment.

## Introduction

Schistosomiasis is a type of zoonotic parasitic disease that is distributed globally and causes serious harm to human health (1). *Schistosoma japonicum* is mainly endemic to China, Indonesia and the Philippines and is a significant public health problem in China (2). The main pathological changes caused by *Schistosoma japonicum* infection are the formation of granulomas and hepatic fibrosis (3). Hepatic fibrosis is the principal cause of serious complications and mortality due to *Schistosoma japonicum* infection (4). Hepatic fibrosis is a compensatory response that is secondary to the process of tissue repair following liver inflammation or damage caused by *Schistosoma japonicum* infection (5). The lack of an endogenous anti-inflammatory and pro-resolving mediator leads to persistent inflammation and causes liver fibrosis (6). Resolvin E1 (RvE1) is a potent anti-inflammatory and pro-resolving member of the E-series resolvins produced from eicosapentaenoic acid (EPA) (7). In the present study, the effects of RvE1 on liver fibrosis in schistosome-infected mice were investigated.

## Materials and methods

### Experimental animals

A total of 30 female Kunming mice (pathogen-free), six-weeks-old and weighing 22±2 g, were purchased from the Jianghan University Animal House (Wuhan, China). The mice were randomized into three groups, with 10 mice per group: Control, model and RvE1 intervention. The model and RvE1 intervention groups were infected with schistosome cercariae through the abdominal skin (20±2 cercariae per mouse). The control mice were not infected. The mice in the RvE1 intervention group were administered 100 ng RvE1 daily, from the day of infection. The RvE1 was dissolved in 2 ml normal saline and administered intragastrically. The mice in the control and model groups received an equivalent volume of normal saline by intragastric administration. Treatment occurred every day for 70 days. Subsequently, all mice were sacrificed for analysis. Blood samples were collected by retro-orbital bleeding. Following opening up of the abdomen, the whole livers were removed. The hepatic middle lobules were harvested and the remaining liver tissue was cryopreserved for the subsequent experiments. Snails infected with schistosome cercariae were obtained from the Hubei Institute of Parasitic Diseases (Wuhan, China), and the surgical instruments and equipment were from the Jianghan University Animal Center. The present study was approved by the Medical Ethics Committee of medical college of Jianghan University (Wuhan, China).

### Measurement of the serum tumor necrosis factor (TNF)-α, interferon (IFN)-γ, alanine aminotransferase (ALT) and aspartate aminotransferase (AST) levels

Serum samples from the individual mice were collected prior to when the mice were sacrificed. The TNF-α and IFN-γ levels were measured using ELISA kits (R&D Systems, Minneapolis, MN, USA). Liver injury was assessed by measuring the serum levels of the liver-associated enzymes ALT and AST using commercially available kits (Shanghai Rongsheng Biotech Co. Ltd., Shanghai, China).

### Liver tissue homogenates

The liver tissue was thawed and rinsed, then cut into sections. The tissue (0.2 g) was placed into a 10-ml beaker. A volume of homogenate solution (0.1 mM Tris-HCl, 0.01 mM EDTA-2Na, 0.01 mM sucrose and 0.8% NaCl) nine-fold (w:v = 1:4) the amount of the tissue was added. The liver was processed with a homogenizer for 6–8 min in order to fully homogenize the sections. The samples were centrifuged at 3,000 × g at 4°C for 10–15 min. Appropriate amounts of clear supernatant liquid were used for the protein detection.

### Laminin (LN), hyaluronic acid (HA), procollagen type III (PC-III) and type IV collagen (IV-C) detection

The LN, HA, PC-III and IV-C concentrations in the serum samples were detected by radioimmunoassays conducted according to the instructions provided with the assay kits. LN, HA, PC-III and IV-C assay kits were provided by Tianjin Atomic Energy Industry (Tianjin, China).

### RNA extraction and reverse transcription-polymerase chain reaction (RT-PCR)

The total RNA of the liver tissues was extracted with TRIzol reagent (Invitrogen Life Technologies, Carlsbad, CA, USA). Two micrograms of the total RNA was used for each reverse transcription reaction for cDNA synthesis. Quantitative PCR was performed using a sequence detector (ABI-Prism StepOnePlus; Applied Biosystems, Foster City, CA, USA) and SYBR Premix Ex Taq [Takara Biotechnology (Dalian) Co., Ltd., Dalian, China] according to the manufacturer’s instructions. PCR was performed using the following primers for TNF-α: 5′-TGAGCACTGAAAGCATGATCC-3′ and 5′-ATCACTCCAAAGTGCAGCAG-3′. A ‘housekeeping’ gene encoding β-non-muscle actin was used as a normalization control. The sequencing involved thermal cycling at 95°C for 1 min (denaturation), 50°C for 1 min (annealing) and 72°C for 1 min (extension). The products were evaluated by agarose gel electrophoresis and analyzed using the ChemiDoc MP imaging system (Bio-Rad, Hercules, CA, USA).

### Western blot analysis

To detect the levels of TNF-α protein, 20 μg protein extracted from the each liver was separated by SDS-PAGE and electroblotted onto a nitrocellulose membrane, which was probed with anti-TNF-α mouse antibody (1:1,000; Cell Signaling Technology, Inc., Beverly, MA, USA) and a polyclonal antibody against glyceraldehyde-3-phosphate dehydrogenase (GAPDH; 1:1,000; Cell Signaling Technology, Beverly, MA, USA). HRP-conjugated goat anti-mouse IgG (1:50,000; Cell Signaling Technology, Beverly, MA, USA) was used to detect the bound mouse antibody, while HRP-conjugated goat anti-rabbit IgG (1:50,000; Cell Signaling Technology, Beverly, MA, USA) was used to detect the anti-GAPDH polyclonal antibody. The secondary antibodies were detected using an ECL Immunoblot Detection system (Pierce Biotechnology, Inc., Rockford, IL, USA).

### Liver pathology

The middle lobule of each liver was fixed in 4% formaldehyde, embedded in paraffin, sectioned at a thickness of 4 μm and placed onto glass slides. The paraffin-embedded samples were dewaxed and stained with hematoxylin and eosin. In order to determine the number and area of the granulomas, five different visual fields were captured at ×400 magnification under a microscope (Olympus, Tokyo, Japan). An image analysis system (VIAS, Ventana Medical Systems, Inc., Tucson, AZ, USA) was applied to measure the area of the granulomas.

### Statistical analysis

The means of triplicate experiments were used for statistical analysis by one-way analysis of variance with post hoc Tukey’s test for pairwise group comparisons (SPSS software, version 13; SPSS, Inc., Chicago, IL, USA). P<0.05 was considered to indicate a statistically significant difference (two-sided).

## Results

### RvE1 treatment improves the liver pathology in infected mice

**Figure 1:**
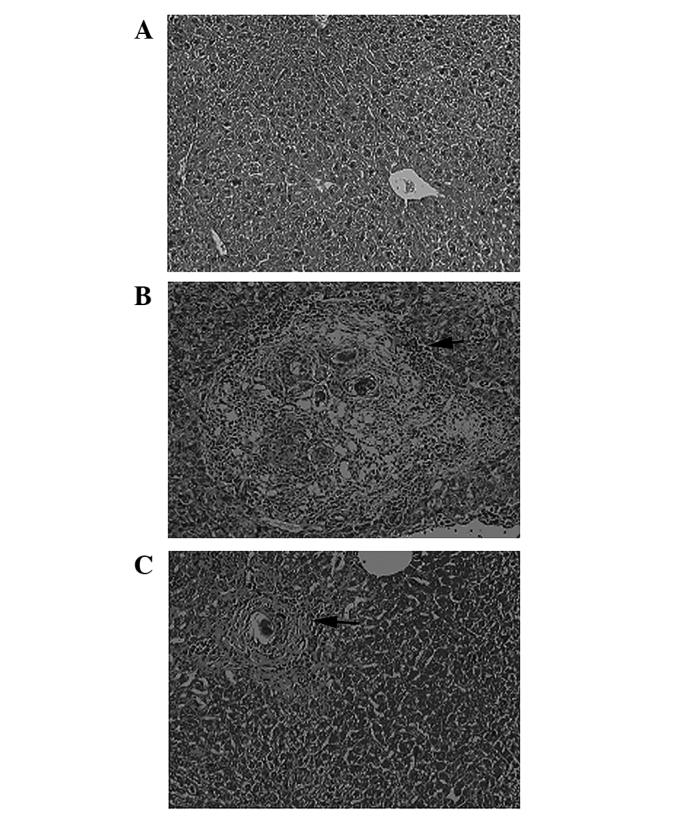
H&E-stained liver tissue from the mice infected with *Schistosoma japonicum* (magnification, ×400). (A) Control group; (B) model group; and (C) RvE1 intervention group. Granuloma formation is present in the model and RvE1 intervention groups. H&E, hematoxylin and eosin; RvE1; Resolvin E1.

The livers from the control mice showed clear lobules and normal structure under microscopic examination. The portal and hepatic sinuses appeared normal with uniform distribution (Fig. 1A). The livers from model mice showed typical damage in the liver lobules. Segregation of the liver by collagen fibers, necrosis lesions in the granulomas and inflammatory cells extensively in the periphery of the granulomas was observed (Fig. 1B). However, the severity of the hepatocellular necrosis and fibroplasia was markedly reduced in the livers of the mice treated with RvE1 compared with that of the model group (Fig. 1C). The mice in the RvE1 intervention group also showed thin fibroseptal attachments and decreased inflammatory cell infiltrations in the livers, demonstrating markedly improved or normal architecture of the hepatic lobules.

### Granuloma number and area are reduced in infected mice receiving RvE1 treatment

The livers from the control mice exhibited no granulomas; therefore, the mean number and area of granulomas in each of the infected groups were significantly increased compared with those of the controls (Table I). However, the mean area of the granulomas in the RvE1 intervention group was smaller than that in the model group.

### RvE1 lowers the levels of markers of liver fibrosis in infected mice

To further measure the extent of the liver fibrosis in the mice, the concentrations of several markers were measured (Table II). LN, HA, PC-III and IV-C concentrations were elevated in each of the infected groups compared with those in the control group. However, in the mice receiving RvE1 treatment, each of these markers exhibited reduced levels compared with those in the model group.

### RvE1 lowers the levels of markers of liver injury in infected mice

To further observe the extent of the liver injury in the mice, the serum concentrations of ALT and AST were measured (Table III). The serum ALT and AST concentrations were increased in each of the infected groups compared with those of the control group. However, the serum ALT and AST concentrations were reduced in the mice that received RvE1 treatment compared with those in the model group.

### RvE1 affects the TNF-a and IFN-γ expression levels in infected mice

**Figure 2:**
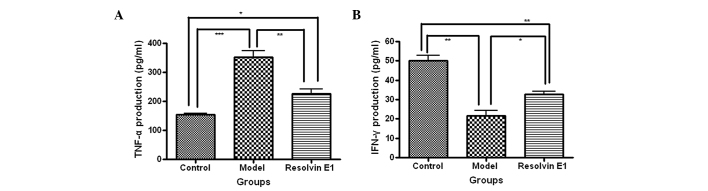
ELISA detection of TNF-α and IFN-γ levels in serum from *Schistosoma japonicum*-infected mice. The (A) TNF-α and (B) and IFN-γ levels in the serum were measured by ELISA assays. Data are presented as the mean ± SD of three independent experiments (^*^P<0.05, ^**^P<0.01, ^***^P<0.001). TNF-α, tumor necrosis factor-α; IFN, interferon; ELISA, enzyme-linked immunosorbent assay.

**Figure 3:**
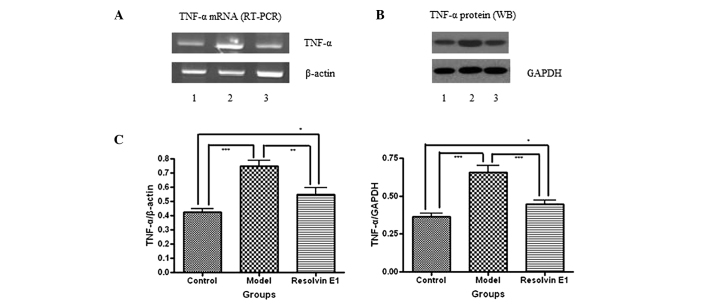
Profiles of TNF-α mRNA (RT-PCR) and protein (western blot analysis) expression levels. (A) The expression levels of TNF-α and β-actin mRNA over time. The 100-bp β-actin mRNA fragment was used as an internal control. (B) The expression levels of TNF-α and GAPDH protein over time. The 37-kDa GAPDH band was used as an internal control. (C) The IOD of TNF-α/β-actin or TNF-α/GAPDH was expressed as the mean ± SD. n=10 at each time (^*^P<0.05, ^**^P<0.01, ^***^P<0.001). TNF-α, tumor necrosis factor-α; β-actin, β-non-muscle actin; GAPDH, glyceraldehyde-3-phosphate dehydrogenase; RT-PCR, reverse transcription polymerase chain reaction; IOD, integral optical density.

The production and expression levels of TNF-α were determined by an ELISA, RT-PCR and western blot analysis. The serum TNF-α production levels were elevated in each of the infected groups compared with those in the control group. However, the serum TNF-α production levels were reduced in the mice receiving RvE1 treatment compared with those in the model group (Fig. 2A). In addition, the TNF-α mRNA and protein levels were also reduced by RvE1 treatment compared with those in the model group (Fig. 3).

The production and expression levels of IFN-γ were determined by ELISA. The serum IFN-γ production levels were reduced in each of the infected groups compared with those in the control group. However, the serum IFN-γ production levels were increased in the mice receiving RvE1 treatment compared with those of the model group (Fig. 2B).

## Discussion

Schistosomiasis is an infectious disease that is seriously harmful to human health (8). *Schistosoma japonicum* is one of the most common public health problem in China (9). *Schistosoma japonicum* is a typical chronic infectious disease that induces hepatic schistosomiasis, and the main pathologic lesions of hepatic schistosomiasis are granuloma formation and liver fibrosis around the schistosome eggs (10). Schistosome eggs are an important source of antigens to which the host is exposed during *Schistosoma japonicum* infection and these cells induce an imbalance in T-cell immunity, which is considered to be a trigger of liver fibrosis (11). The imbalance of T helper (Th)1/Th2 cell immunity excessively activates Th2 and induces hematopoietic stem cells to differentiate into fibroblasts (7). These fibroblasts increase the levels of collagen formation and suppress its decomposition, which eventually results in matrix protein deposition and fibrosis (12). Th2-derived cytokines cause liver damage and proliferation of fibrous tissue, accelerating fibrosis through the activation of macrophages and the induction of TNF-α and other inflammatory cytokines (13). There is a correlation between elevated serum levels of TNF-α and an increased degree of liver fibrosis, and TNF-α generally promotes liver fibrosis. In addition, IFN-γ is associated with anti-hepatic fibrosis, which is a very strong anti-fibrotic factor. These results have been demonstrated in numerous studies of the induction of liver fibrosis in models with hepatic schistosomiasis (14–16). The inflammatory cytokines produced in hepatic schistosomiasis induce liver fibrosis to protect the liver.

RvE1 (5S,12R,18R-trihydroxy-6Z,8E,10E,14Z,16E-eicosapentaenoic acid) is an endogenous anti-inflammatory and pro-resolving mediator derived from the ω-3 fatty acid EPA during resolution (17). Systemic aspirin treatment enhances local exudate conversion of EPA to the potent, bioactive RvE1 (18). RvE1 stimulates endogenous resolution mechanisms *in vivo* in complex disease models and *in vitro* (19,20). In nanogram quantities RvE1 promotes resolution of acute inflammation by regulating leukocyte infiltration, increasing the ingestion of apoptotic neutrophils by macrophages and enhancing the clearance of phagocytes to the lymph nodes and spleen (21). In the present study, the effects of administered RvE1 were investigated and it was demonstrated that RvE1 regulates the levels of inflammatory factors by an anti-inflammatory and pro-resolving mechanism, improves the local inflammatory response and effectively alleviates liver fibrosis in schistosome-infected mice. The serum levels of TNF-α were reduced in the RvE1-treated mice compared with those in the untreated infected mice. Similarly, the mRNA and protein expression levels of TNF-α were reduced in the RvE1-treated mice compared with those in the untreated infected mice. The serum levels of IFN-γ were increased in the infected animals treated with RvE1 compared with those in the untreated infected mice. The results suggest that the RvE1 treatment changed the cytokine levels and thereby resolved the inflammation. This anti-inflammatory response may be responsible for the less severe liver pathology observed in the infected mice treated with RvE1 compared with that in the untreated infected mice. The levels of serum ALT and AST were significantly reduced following the RvE1 treatment compared with those in the untreated infected mice, which indicated that the RvE1-treated mice exhibited attenuated liver injury. Thus, it is hypothesized that interventions in the inflammatory response may effectively alleviate liver injury.

The effects of administered RvE1 on liver pathology in schistosome-infected mice were also investigated. Administered RvE1 treatment reduced the effects of schistosome infection on the liver. While granuloma formation occurred with the same frequency as in the untreated infected mice, the area of the granulomas was significantly reduced in the RvE1-treated mice. In addition, the concentrations of the serum indicators of liver fibrosis (LN, HA, PC-III and IV-C) were significantly lower in the infected mice treated with RvE1 than those in the untreated infected mice. These results further confirmed the anti-fibrotic effects of the administration of RvE1.

In conclusion, RvE1 treatment regulates the levels of cytokines to reduce the inflammatory response within the liver in order to resist fibrosis following schistosome infection. The present study suggests that administered RvE1 treatment may slow the progression of liver fibrosis in individuals affected by schistosomiasis.

## Figures and Tables

**Table I:** Granuloma numbers and area in animal models of *Schistosoma japonicum* untreated or treated with RvE1 (n=10 per group).

Group	Mean no. of granulomas	Mean area of granuloma (mm^2^)
Control	0.0±0.00	0.00±0.00
Model	11.94±2.87^a^	17.04±3.51^a^
RvE1	10.42±2.47^a^	10.26±4.38^a^,^b^
F-value	88.44	82.99
P-value	0.00	0.00

a P<0.05 vs. the control group;

b P<0.05 vs. the model group.

Data are presented as the mean ±SD. RvE1, resolvin E1.

**Table II:** Concentrations of LN, HA, PC-III and IV-C in serum samples from RvE1-treated and untreated mice (n=10 per group).

Group	LN (ng/ml)	HA (ng/ml)	PC-III (ng/ml)	IV-C (ng/ml)
Control	10434.6±67.3	34.5±6.3	59.2±11.7	72.2±17.2
Model	10741.5±124.2^a^	65.8±15.7^a^	134.4±31.4^a^	139.2±35.2^a^
RvE1	10554.8±149.8^a^,^b^	44.4±8.6^a^,^b^	76.7±18.2^a^,^b^	88.2±15.8^a^,^b^
F-value	18.01	20.41	34.05	19.75
P-value	0.00	0.00	0.00	0.00

a P<0.05 vs. the control group;

b P<0.05 vs. the model group.

Data are presented as the mean ±SD. LN, laminin; HA, hyaluronic acid; PC-III, procollagen type III; IV-C, type IV collagen. RvE1, resolvin E1.

**Table III:** Effect of treatment with RvE1 on the levels of serum transaminases (ALT/AST) in a *Schistosoma japonicum*-induced liver fibrosis model (n=10 per group).

Group	ALT (U/l)	AST (U/l)
Control	37.42±7.52	41.92±8.30
Model	358.08±91.57^a^	380.89±88.22^a^
RvE1	98.70±43.77^a^,^b^	153.85±39.29^a^,^b^
F-value	91.92	90.21
P-value	0.00	0.00

a P<0.05 vs. the control group;

b P<0.05 vs. the model group.

Data are presented as the mean ± SD. RvE1, resolvin E1; ALT, alanine aminotransferase; AST, aspartate aminotransferase.
